# Supplementary material for: A Comparative Study of Nine SARS-CoV-2 IgG Lateral Flow Assays Using Both Post-Infection and Post-Vaccination Samples
Source: J Clin Med. 2022 Apr 8;11(8):2100. doi: 10.3390/jcm11082100 (PMC9032267; doi:10.3390/jcm11082100)
Supplement: Supplementary file 1 [file jcm-11-02100-s001.zip › jcm-1628005-supplementary.pdf]

Supplementary File S1: Regression Models Results
Generalized linear model (logistic regression)

The generalized linear models are here to confirm the linear fitting of tests results. Gaussian distribution was chosen with logit identity to build normal regression model. Finally, the chi-squared test (resulting from ANOVA) is performed to validate the goodness of fit of the given variables.

Essentially, these steps will allow to define whether we have a linear response between Test results (as numeric values: Negative=0, weak positive=1 and positive=2), measured IgG concentration, and the other measured variables.

By separating the tests by Test brands and by type of vaccine, we can validate weather in the given condition we should get a valid test result and if the other variables have an impact.

Analysis per Test name and vaccine

Vaccine: Pfizer

Vaccine: Pfizer Test: Abnova

Linear regression of Abnova, Pfizer: Test.Result.num ~ S1.IgG + Date.diffTest + Gender + Age, R2=0.168

	Estimate	Std. Error	t value	Pr(>|t|)
(Intercept)	−0.144	0.826	−0.174	0.865
S1.IgG	0.035	0.063	0.563	0.584
Date.diffTest	−0.007	0.006	−1.074	0.306
GenderM	−0.147	0.370	−0.398	0.698
Age	0.007	0.018	0.389	0.705

Anova of the linear model

	Df	Deviance	Resid. Df	Resid. Dev	Pr(>Chi)
NULL	NA	NA	15	3.0	NA
S1.IgG	1	0.079	14	2.9	0.55
Date.diffTest	1	0.384	13	2.5	0.19
Gender	1	0.008	12	2.5	0.85
Age	1	0.034	11	2.5	0.70

Vaccine: Pfizer Test: Nadal

Test results not diverse.

Vaccine: Pfizer Test: Ring.Biotech

Linear regression of Ring.Biotech, Pfizer: Test.Result.num ~ S1.IgG + Date.diffTest + Gender + Age, R2=0.719

	Estimate	Std. Error	t value	Pr(>|t|)
(Intercept)	−0.9180	0.3110	−2.9540	0.0093
S1.IgG	0.0420	0.0240	1.7510	0.0990
Date.diffTest	0.0000	0.0030	−0.1270	0.9006
GenderM	0.4150	0.1380	3.0070	0.0084
Age	0.0140	0.0060	2.2800	0.0366

Anova of the linear model

	Df	Deviance	Resid. Df	Resid. Dev	Pr(>Chi)
NULL	NA	NA	20	2.57	NA
S1.IgG	1	0.19	19	2.39	4.3e−02
Date.diffTest	1	0.02	18	2.37	5.0e−01
Gender	1	1.41	17	0.96	2.3e−08
Age	1	0.23	16	0.72	2.3e−02

Vaccine: Pfizer Test: Wondfo

Linear regression of Wondfo, Pfizer: Test.Result.num ~ S1.IgG + Date.diffTest + Gender + Age, R2=0.663

	Estimate	Std. Error	t value	Pr(>|t|)
(Intercept)	−0.17400	0.77100	−0.22600	0.82437
S1.IgG	0.30400	0.06000	5.04900	0.00012
Date.diffTest	0.00800	0.00600	1.36500	0.19104
GenderM	0.52000	0.34200	1.52000	0.14796
Age	−0.01900	0.01500	−1.26000	0.22573

Anova of the linear model

	Df	Deviance	Resid. Df	Resid. Dev	Pr(>Chi)
NULL	NA	NA	20	13.1	NA
S1.IgG	1	7.12	19	6.0	4.0e−07
Date.diffTest	1	0.90	18	5.1	7.2e−02
Gender	1	0.25	17	4.9	3.4e−01
Age	1	0.44	16	4.4	2.1e−01

Vaccine: Pfizer Test: Labnovation

Test results not diverse.

Vaccine: Pfizer Test: Biosynex

Linear regression of Biosynex, Pfizer: Test.Result.num ~ S1.IgG + Date.diffTest + Gender + Age, R2=0.842

	Estimate	Std. Error	t value	Pr(>|t|)
(Intercept)	−8.3e−01	4.2e−01	−2.0e+00	6.6e−02
S1.IgG	2.8e−01	3.3e−02	8.4e+00	2.8e−07
Date.diffTest	5.0e−03	3.0e−03	1.5e+00	1.7e−01
GenderM	−2.6e−01	1.9e−01	−1.4e+00	1.8e−01
Age	1.1e−02	8.0e−03	1.3e+00	2.2e−01

Anova of the linear model

	Df	Deviance	Resid. Df	Resid. Dev	Pr(>Chi)
NULL	NA	NA	20	8.3	NA
S1.IgG	1	6.69	19	1.6	1.4e−19
Date.diffTest	1	0.10	18	1.5	2.6e−01
Gender	1	0.05	17	1.4	4.3e−01
Age	1	0.14	16	1.3	2.0e−01

Vaccine: Pfizer Test: Dynamiker

Linear regression of Dynamiker, Pfizer: Test.Result.num ~ S1.IgG + Date.diffTest + Gender + Age, R2=0.363

	Estimate	Std. Error	t value	Pr(>|t|)
(Intercept)	−0.701	0.605	−1.160	0.263
S1.IgG	0.086	0.047	1.815	0.088
Date.diffTest	−0.004	0.005	−0.887	0.388
GenderM	0.315	0.268	1.172	0.258
Age	0.008	0.012	0.692	0.499

Anova of the linear model

	Df	Deviance	Resid. Df	Resid. Dev	Pr(>Chi)
NULL	NA	NA	20	4.3	NA
S1.IgG	1	0.575	19	3.7	0.067
Date.diffTest	1	0.199	18	3.5	0.280
Gender	1	0.699	17	2.8	0.043
Age	1	0.082	16	2.7	0.489

Vaccine: Pfizer Test: Cortez

Linear regression of Cortez, Pfizer: Test.Result.num ~ S1.IgG + Date.diffTest + Gender + Age, R2=0.466

	Estimate	Std. Error	t value	Pr(>|t|)
(Intercept)	−0.8730	0.8080	−1.0800	0.2963
S1.IgG	0.1860	0.0630	2.9440	0.0095
Date.diffTest	−0.0020	0.0070	−0.3330	0.7433
GenderM	−0.2870	0.3590	−0.8000	0.4352
Age	0.0260	0.0160	1.6140	0.1260

Anova of the linear model

	Df	Deviance	Resid. Df	Resid. Dev	Pr(>Chi)
NULL	NA	NA	20	9.1	NA
S1.IgG	1	3.252	19	5.9	0.0011
Date.diffTest	1	0.197	18	5.7	0.4222
Gender	1	0.018	17	5.7	0.8082
Age	1	0.795	16	4.9	0.1064

Vaccine: Pfizer Test: CTK

Test results not diverse.

Vaccine: Moderna

Vaccine: Moderna Test: Abnova

Test results not diverse.

Vaccine: Moderna Test: Nadal

Linear regression of Nadal, Moderna: Test.Result.num ~ S1.IgG + Date.diffTest + Gender + Age, R2=0.766

	Estimate	Std. Error	t value	Pr(>|t|)
(Intercept)	−2.8e−01	4.3e−01	−6.5e−01	5.2e−01
S1.IgG	2.6e−01	3.5e−02	7.2e+00	4.1e−07
Date.diffTest	5.0e−03	2.0e−03	2.5e+00	2.3e−02
GenderM	9.4e−02	1.8e−01	5.3e−01	6.0e−01
Age	0.0e+00	5.0e−03	−7.3e−02	9.4e−01

Anova of the linear model

	Df	Deviance	Resid. Df	Resid. Dev	Pr(>Chi)
NULL	NA	NA	25	11.5	NA

	Df	Deviance	Resid. Df	Resid. Dev	Pr(>Chi)
S1.IgG	1	7.95362	24	3.6	3.7e−15
Date.diffTest	1	0.84433	23	2.7	1.0e−02
Gender	1	0.03843	22	2.7	5.8e−01
Age	1	0.00068	21	2.7	9.4e−01

Vaccine: Moderna Test: Ring.Biotech

Test results not diverse.

Vaccine: Moderna Test: Wondfo

Linear regression of Wondfo, Moderna: Test.Result.num ~ S1.IgG + Date.diffTest + Gender + Age, R2=0.754

	Estimate	Std. Error	t value	Pr(>|t|)
(Intercept)	−3.4e−01	5.6e−01	−6.1e−01	5.5e−01
S1.IgG	2.6e−01	4.5e−02	5.7e+00	1.2e−05
Date.diffTest	−1.0e−03	3.0e−03	−3.8e−01	7.1e−01
GenderM	−3.8e−01	2.3e−01	−1.7e+00	1.1e−01
Age	3.0e−03	6.0e−03	4.6e−01	6.5e−01

Anova of the linear model

	Df	Deviance	Resid. Df	Resid. Dev	Pr(>Chi)
NULL	NA	NA	25	17.9	NA
S1.IgG	1	12.745	24	5.1	6.4e−15
Date.diffTest	1	0.063	23	5.1	5.8e−01
Gender	1	0.628	22	4.4	8.4e−02
Age	1	0.045	21	4.4	6.4e−01

Vaccine: Moderna Test: Labnovation

Test results not diverse.

Vaccine: Moderna Test: Biosynex

Test results not diverse.

Vaccine: Moderna Test: Dynamiker

Test results not diverse.

Vaccine: Moderna Test: Cortez

Linear regression of Cortez, Moderna: Test.Result.num ~ S1.IgG + Date.diffTest + Gender + Age, R2=0.786

	Estimate	Std. Error	t value	Pr(>|t|)
(Intercept)	−6.0e−01	5.0e−01	−1.2e+00	2.4e−01
S1.IgG	2.9e−01	4.0e−02	7.1e+00	4.9e−07
Date.diffTest	−3.0e−03	3.0e−03	−1.2e+00	2.5e−01
GenderM	2.4e−02	2.0e−01	1.2e−01	9.1e−01
Age	5.0e−03	6.0e−03	9.3e−01	3.6e−01

Anova of the linear model

	Df	Deviance	Resid. Df	Resid. Dev	Pr(>Chi)
NULL	NA	NA	25	16.5	NA
S1.IgG	1	1.3e+01	24	3.9	4.3e−18
Date.diffTest	1	1.9e−01	23	3.7	2.9e−01
Gender	1	3.0e−06	22	3.7	1.0e+00
Age	1	1.4e−01	21	3.5	3.5e−01

Vaccine: Moderna Test: CTK

Test results not diverse.

Vaccine: SinoVac

Vaccine: SinoVac Test: Abnova

Test results not diverse.

Vaccine: SinoVac Test: Nadal

Linear regression of Nadal, SinoVac: Test.Result.num ~ S1.IgG + Date.diffTest + Gender + Age, R2=0.694

	Estimate	Std. Error	t value	Pr(>|t|)
(Intercept)	−5.1e−02	4.8e−01	−1.1e−01	9.1e−01
S1.IgG	3.2e−01	3.1e−02	1.0e+01	1.2e−14
Date.diffTest	5.0e−03	5.0e−03	1.1e+00	2.6e−01
GenderM	−2.6e−01	1.4e−01	−1.9e+00	6.5e−02
Age	−6.0e−03	8.0e−03	−7.2e−01	4.8e−01

Anova of the linear model

	Df	Deviance	Resid. Df	Resid. Dev	Pr(>Chi)
NULL	NA	NA	63	57	NA

	Df	Deviance	Resid. Df	Resid. Dev	Pr(>Chi)
S1.IgG	1	38.20	62	19	5.7e−30
Date.diffTest	1	0.17	61	19	4.4e−01
Gender	1	1.03	60	18	6.1e−02
Age	1	0.15	59	17	4.7e−01

Vaccine: SinoVac Test: Ring.Biotech

Linear regression of Ring.Biotech, SinoVac: Test.Result.num ~ S1.IgG + Date.diffTest + Gender + Age, R2=0.491

	Estimate	Std. Error	t value	Pr(>|t|)
(Intercept)	2.8e−02	5.0e−01	5.6e−02	9.6e−01
S1.IgG	2.1e−01	3.2e−02	6.5e+00	2.0e−08
Date.diffTest	2.0e−03	5.0e−03	5.0e−01	6.2e−01
GenderM	−9.1e−02	1.5e−01	−6.2e−01	5.4e−01
Age	−9.0e−03	9.0e−03	−1.1e+00	2.9e−01

Anova of the linear model

	Df	Deviance	Resid. Df	Resid. Dev	Pr(>Chi)
NULL	NA	NA	63	38	NA
S1.IgG	1	18.120	62	20	1.0e−13
Date.diffTest	1	0.052	61	20	6.9e−01
Gender	1	0.120	60	20	5.5e−01
Age	1	0.370	59	19	2.9e−01

Vaccine: SinoVac Test: Wondfo

Linear regression of Wondfo, SinoVac: Test.Result.num ~ S1.IgG + Date.diffTest + Gender + Age, R2=0.565

	Estimate	Std. Error	t value	Pr(>|t|)
(Intercept)	4.2e−01	5.1e−01	8.2e−01	4.1e−01
S1.IgG	2.3e−01	3.3e−02	7.0e+00	2.4e−09
Date.diffTest	−4.0e−03	5.0e−03	−8.4e−01	4.1e−01
GenderM	−1.5e−01	1.5e−01	−1.0e+00	3.1e−01
Age	−4.0e−03	9.0e−03	−4.2e−01	6.7e−01

Anova of the linear model

	Df	Deviance	Resid. Df	Resid. Dev	Pr(>Chi)
NULL	NA	NA	63	45	NA
S1.IgG	1	24.83	62	21	7.1e−18

	Df	Deviance	Resid. Df	Resid. Dev	Pr(>Chi)
Date.diffTest	1	0.39	61	20	2.8e−01
Gender	1	0.34	60	20	3.1e−01
Age	1	0.06	59	20	6.7e−01

Vaccine: SinoVac Test: Labnovation

Linear regression of Labnovation, SinoVac: Test.Result.num ~ S1.IgG + Date.diffTest + Gender + Age, R2=0.602

	Estimate	Std. Error	t value	Pr(>|t|)
(Intercept)	2.8e−01	5.4e−01	5.2e−01	6.1e−01
S1.IgG	2.8e−01	3.5e−02	8.2e+00	2.8e−11
Date.diffTest	2.0e−03	5.0e−03	3.9e−01	7.0e−01
GenderM	−6.2e−02	1.6e−01	−3.9e−01	7.0e−01
Age	−9.0e−03	9.0e−03	−9.6e−01	3.4e−01

Anova of the linear model

	Df	Deviance	Resid. Df	Resid. Dev	Pr(>Chi)
NULL	NA	NA	63	55	NA
S1.IgG	1	32.642	62	22	6.8e−21
Date.diffTest	1	0.042	61	22	7.4e−01
Gender	1	0.054	60	22	7.0e−01
Age	1	0.341	59	22	3.4e−01

Vaccine: SinoVac Test: Biosynex

Linear regression of Biosynex, SinoVac: Test.Result.num ~ S1.IgG + Date.diffTest + Gender + Age, R2=0.771

	Estimate	Std. Error	t value	Pr(>|t|)
(Intercept)	−7.7e−01	3.9e−01	−2.0e+00	5.4e−02
S1.IgG	3.3e−01	2.5e−02	1.3e+01	4.5e−19
Date.diffTest	8.0e−03	4.0e−03	2.2e+00	3.4e−02
GenderM	−1.6e−01	1.2e−01	−1.4e+00	1.6e−01
Age	1.0e−03	7.0e−03	8.0e−02	9.4e−01

Anova of the linear model

	Df	Deviance	Resid. Df	Resid. Dev	Pr(>Chi)
NULL	NA	NA	63	51	NA
S1.IgG	1	38.1748	62	13	5.9e−44
Date.diffTest	1	0.7220	61	12	5.6e−02

	Df	Deviance	Resid. Df	Resid. Dev	Pr(>Chi)
Gender	1	0.3909	60	12	1.6e−01
Age	1	0.0013	59	12	9.4e−01

Vaccine: SinoVac Test: Dynamiker

Linear regression of Dynamiker, SinoVac: Test.Result.num ~ S1.IgG + Date.diffTest + Gender + Age, R2=0.593

	Estimate	Std. Error	t value	Pr(>|t|)
(Intercept)	6.1e−02	4.9e−01	1.2e−01	9.0e−01
S1.IgG	2.5e−01	3.2e−02	7.8e+00	1.4e−10
Date.diffTest	−1.0e−03	5.0e−03	−2.6e−01	7.9e−01
GenderM	−1.8e−01	1.4e−01	−1.3e+00	2.0e−01
Age	−2.0e−03	8.0e−03	−2.9e−01	7.7e−01

Anova of the linear model

	Df	Deviance	Resid. Df	Resid. Dev	Pr(>Chi)
NULL	NA	NA	63	45	NA
S1.IgG	1	26.187	62	19	5.2e−20
Date.diffTest	1	0.095	61	19	5.8e−01
Gender	1	0.512	60	18	2.0e−01
Age	1	0.026	59	18	7.7e−01

Vaccine: SinoVac Test: Cortez

Linear regression of Cortez, SinoVac: Test.Result.num ~ S1.IgG + Date.diffTest + Gender + Age, R2=0.541

	Estimate	Std. Error	t value	Pr(>|t|)
(Intercept)	1.4e+00	5.3e−01	2.7e+00	9.4e−03
S1.IgG	2.0e−01	3.4e−02	6.0e+00	1.3e−07
Date.diffTest	−1.0e−02	5.0e−03	−1.9e+00	6.0e−02
GenderM	2.2e−02	1.5e−01	1.4e−01	8.9e−01
Age	−1.0e−02	9.0e−03	−1.1e+00	2.9e−01

Anova of the linear model

	Df	Deviance	Resid. Df	Resid. Dev	Pr(>Chi)
NULL	NA	NA	63	46	NA
S1.IgG	1	23.2673	62	23	8.9e−16
Date.diffTest	1	1.3143	61	22	5.6e−02
Gender	1	0.0086	60	22	8.8e−01

	Df	Deviance	Resid. Df	Resid. Dev	Pr(>Chi)
Age	1	0.4141	59	21	2.8e−01

Vaccine: SinoVac Test: CTK

Linear regression of CTK, SinoVac: Test.Result.num ~ S1.IgG + Date.diffTest + Gender + Age, R2=0.731

	Estimate	Std. Error	t value	Pr(>|t|)
(Intercept)	−1.2e−01	4.2e−01	−2.8e−01	7.8e−01
S1.IgG	3.1e−01	2.7e−02	1.1e+01	1.8e−16
Date.diffTest	5.0e−03	4.0e−03	1.2e+00	2.3e−01
GenderM	−1.4e−01	1.2e−01	−1.1e+00	2.8e−01
Age	−5.0e−03	7.0e−03	−6.4e−01	5.3e−01

Anova of the linear model

	Df	Deviance	Resid. Df	Resid. Dev	Pr(>Chi)
NULL	NA	NA	63	51	NA
S1.IgG	1	36.667	62	14	3.4e−36
Date.diffTest	1	0.238	61	14	3.1e−01
Gender	1	0.277	60	14	2.8e−01
Age	1	0.094	59	14	5.2e−01

Vaccine: None

Vaccine: None Test: Abnova

Test results not diverse.

Vaccine: None Test: Nadal

Linear regression of Nadal, None: Test.Result.num ~ S1.IgG + Date.diffTest + Gender + Age, R2=0.537

	Estimate	Std. Error	t value	Pr(>|t|)
(Intercept)	8.7e−01	6.1e−01	1.4e+00	1.7e−01
S1.IgG	1.7e−01	3.1e−02	5.3e+00	9.9e−06
Date.diffTest	−6.0e−03	6.0e−03	−9.2e−01	3.7e−01
GenderM	3.0e−01	2.7e−01	1.1e+00	2.8e−01
Age	−4.0e−03	9.0e−03	−5.0e−01	6.2e−01

Anova of the linear model

	Df	Deviance	Resid. Df	Resid. Dev	Pr(>Chi)
NULL	NA	NA	33	22	NA

	Df	Deviance	Resid. Df	Resid. Dev	Pr(>Chi)
S1.IgG	1	10.887	32	11	2.8e−08
Date.diffTest	1	0.440	31	11	2.6e−01
Gender	1	0.470	30	10	2.5e−01
Age	1	0.087	29	10	6.2e−01

Vaccine: None Test: Ring.Biotech

Linear regression of Ring.Biotech, None: Test.Result.num ~ S1.IgG + Date.diffTest + Gender + Age, R2=0.296

	Estimate	Std. Error	t value	Pr(>|t|)
(Intercept)	1.723	0.832	2.070	0.047
S1.IgG	0.115	0.042	2.734	0.011
Date.diffTest	−0.014	0.008	−1.619	0.116
GenderM	−0.194	0.368	−0.527	0.602
Age	−0.002	0.012	−0.186	0.854

Anova of the linear model

	Df	Deviance	Resid. Df	Resid. Dev	Pr(>Chi)
NULL	NA	NA	33	27	NA
S1.IgG	1	6.102	32	21	0.0021
Date.diffTest	1	1.584	31	19	0.1174
Gender	1	0.169	30	19	0.6093
Age	1	0.022	29	19	0.8526

Vaccine: None Test: Wondfo

Linear regression of Wondfo, None: Test.Result.num ~ S1.IgG + Date.diffTest + Gender + Age, R2=0.083

	Estimate	Std. Error	t value	Pr(>|t|)
(Intercept)	0.235	0.776	0.303	0.764
S1.IgG	0.007	0.039	0.173	0.864
Date.diffTest	−0.004	0.008	−0.570	0.573
GenderM	0.048	0.343	0.141	0.889
Age	0.016	0.011	1.493	0.146

Anova of the linear model

	Df	Deviance	Resid. Df	Resid. Dev	Pr(>Chi)
NULL	NA	NA	33	18	NA
S1.IgG	1	6.2e−02	32	18	0.74

	Df	Deviance	Resid. Df	Resid. Dev	Pr(>Chi)
Date.diffTest	1	1.6e−01	31	18	0.59
Gender	1	6.3e−06	30	18	1.00
Age	1	1.3e+00	29	16	0.14

Vaccine: None Test: Labnovation

Linear regression of Labnovation, None: Test.Result.num ~ S1.IgG + Date.diffTest + Gender + Age, R2=0.247

	Estimate	Std. Error	t value	Pr(>|t|)
(Intercept)	1.8700	0.6720	2.7820	0.0094
S1.IgG	0.0870	0.0340	2.5560	0.0161
Date.diffTest	−0.0080	0.0070	−1.1450	0.2617
GenderM	0.0440	0.2970	0.1480	0.8832
Age	−0.0050	0.0090	−0.5390	0.5941

Anova of the linear model

	Df	Deviance	Resid. Df	Resid. Dev	Pr(>Chi)
NULL	NA	NA	33	16	NA
S1.IgG	1	3.259	32	13	0.0054
Date.diffTest	1	0.610	31	12	0.2289
Gender	1	0.017	30	12	0.8406
Age	1	0.122	29	12	0.5900

Vaccine: None Test: Biosynex

Linear regression of Biosynex, None: Test.Result.num ~ S1.IgG + Date.diffTest + Gender + Age, R2=0.484

	Estimate	Std. Error	t value	Pr(>|t|)
(Intercept)	3.0e−01	7.0e−01	4.4e−01	6.7e−01
S1.IgG	1.7e−01	3.5e−02	5.0e+00	2.8e−05
Date.diffTest	−1.0e−03	7.0e−03	−1.8e−01	8.6e−01
GenderM	3.8e−01	3.1e−01	1.2e+00	2.3e−01
Age	0.0e+00	1.0e−02	−2.5e−02	9.8e−01

Anova of the linear model

	Df	Deviance	Resid. Df	Resid. Dev	Pr(>Chi)
NULL	NA	NA	33	26	NA
S1.IgG	1	1.2e+01	32	14	4.3e−07
Date.diffTest	1	6.1e−02	31	14	7.1e−01

	Df	Deviance	Resid. Df	Resid. Dev	Pr(>Chi)
Gender	1	6.9e−01	30	13	2.2e−01
Age	1	2.7e−04	29	13	9.8e−01

Vaccine: None Test: Dynamiker

Linear regression of Dynamiker, None: Test.Result.num ~ S1.IgG + Date.diffTest + Gender + Age, R2=0.254

	Estimate	Std. Error	t value	Pr(>|t|)
(Intercept)	0.8390	0.7960	1.0540	0.3007
S1.IgG	0.1130	0.0400	2.8110	0.0088
Date.diffTest	−0.0050	0.0080	−0.6810	0.5014
GenderM	0.2120	0.3520	0.6040	0.5505
Age	−0.0030	0.0110	−0.2730	0.7865

Anova of the linear model

	Df	Deviance	Resid. Df	Resid. Dev	Pr(>Chi)
NULL	NA	NA	33	23	NA
S1.IgG	1	5.176	32	18	0.0031
Date.diffTest	1	0.371	31	17	0.4283
Gender	1	0.237	30	17	0.5265
Age	1	0.044	29	17	0.7846

Vaccine: None Test: Cortez

Linear regression of Cortez, None: Test.Result.num ~ S1.IgG + Date.diffTest + Gender + Age, R2=0.307

	Estimate	Std. Error	t value	Pr(>|t|)
(Intercept)	1.3560	0.6860	1.9770	0.0576
S1.IgG	0.1120	0.0350	3.2230	0.0031
Date.diffTest	−0.0060	0.0070	−0.9110	0.3698
GenderM	−0.1560	0.3030	−0.5150	0.6107
Age	−0.0010	0.0100	−0.1090	0.9139

Anova of the linear model

	Df	Deviance	Resid. Df	Resid. Dev	Pr(>Chi)
NULL	NA	NA	33	18	NA
S1.IgG	1	5.2157	32	13	0.00057
Date.diffTest	1	0.3157	31	13	0.39650
Gender	1	0.1126	30	13	0.61255

	Df	Deviance	Resid. Df	Resid. Dev	Pr(>Chi)
Age	1	0.0052	29	13	0.91320

Vaccine: None Test: CTK

Linear regression of CTK, None: Test.Result.num ~ S1.IgG + Date.diffTest + Gender + Age, R2=0.463

	Estimate	Std. Error	t value	Pr(>|t|)
(Intercept)	3.6e−01	7.5e−01	4.8e−01	6.3e−01
S1.IgG	1.8e−01	3.8e−02	4.9e+00	3.8e−05
Date.diffTest	−2.0e−03	8.0e−03	−2.1e−01	8.4e−01
GenderM	1.4e−01	3.3e−01	4.2e−01	6.8e−01
Age	0.0e+00	1.1e−02	−3.8e−02	9.7e−01

Anova of the linear model

	Df	Deviance	Resid. Df	Resid. Dev	Pr(>Chi)
NULL	NA	NA	33	28	NA
S1.IgG	1	1.3e+01	32	15	6.7e−07
Date.diffTest	1	4.0e−02	31	15	7.8e−01
Gender	1	9.4e−02	30	15	6.7e−01
Age	1	7.4e−04	29	15	9.7e−01

Analysis per Test name and Immune type

Vaccine: RNA

Vaccine: RNA Test: Abnova

Test results not diverse.

Vaccine: RNA Test: Nadal

Linear regression of Nadal, RNA: Test.Result.num ~ S1.IgG + Date.diffTest + Gender + Age, R2=0.651

	Estimate	Std. Error	t value	Pr(>|t|)
(Intercept)	4.3e−01	2.9e−01	1.5e+00	1.4e−01
S1.IgG	2.0e−01	2.6e−02	7.7e+00	1.6e−09
Date.diffTest	3.0e−03	2.0e−03	1.8e+00	8.4e−02
GenderM	−1.8e−01	1.2e−01	−1.4e+00	1.6e−01
Age	−2.0e−03	4.0e−03	−4.5e−01	6.5e−01

Anova of the linear model

	Df	Deviance	Resid. Df	Resid. Dev	Pr(>Chi)
NULL	NA	NA	46	16.4	NA
S1.IgG	1	9.766	45	6.7	2.8e−17
Date.diffTest	1	0.572	44	6.1	4.1e−02
Gender	1	0.323	43	5.8	1.2e−01
Age	1	0.028	42	5.7	6.5e−01

Vaccine: RNA Test: Ring.Biotech

Linear regression of Ring.Biotech, RNA: Test.Result.num ~ S1.IgG + Date.diffTest + Gender + Age, R2=0.278

	Estimate	Std. Error	t value	Pr(>|t|)
(Intercept)	−0.4400	0.1700	−2.5870	0.0132
S1.IgG	0.0350	0.0150	2.2460	0.0301
Date.diffTest	−0.0010	0.0010	−0.6750	0.5031
GenderM	0.2180	0.0730	2.9770	0.0048
Age	0.0040	0.0020	1.6970	0.0971

Anova of the linear model

	Df	Deviance	Resid. Df	Resid. Dev	Pr(>Chi)
NULL	NA	NA	46	2.8	NA
S1.IgG	1	0.096	45	2.7	0.159
Date.diffTest	1	0.024	44	2.7	0.479
Gender	1	0.521	43	2.2	0.001
Age	1	0.139	42	2.0	0.090

Vaccine: RNA Test: Wondfo

Linear regression of Wondfo, RNA: Test.Result.num ~ S1.IgG + Date.diffTest + Gender + Age, R2=0.654

	Estimate	Std. Error	t value	Pr(>|t|)
(Intercept)	−7.6e−01	3.9e−01	−1.9e+00	5.9e−02
S1.IgG	3.0e−01	3.6e−02	8.3e+00	1.9e−10
Date.diffTest	3.0e−03	3.0e−03	1.2e+00	2.4e−01
GenderM	−3.5e−02	1.7e−01	−2.1e−01	8.4e−01
Age	0.0e+00	6.0e−03	−1.6e−02	9.9e−01

Anova of the linear model

	Df	Deviance	Resid. Df	Resid. Dev	Pr(>Chi)
NULL	NA	NA	46	31	NA
S1.IgG	1	2.0e+01	45	11	1.2e−18
Date.diffTest	1	4.5e−01	44	11	1.9e−01
Gender	1	1.2e−02	43	11	8.3e−01
Age	1	6.8e−05	42	11	9.9e−01

Vaccine: RNA Test: Labnovation

Linear regression of Labnovation, RNA: Test.Result.num ~ S1.IgG + Date.diffTest + Gender + Age, R2=0.59

	Estimate	Std. Error	t value	Pr(>|t|)
(Intercept)	3.6e−01	3.3e−01	1.1e+00	2.8e−01
S1.IgG	2.0e−01	3.0e−02	6.5e+00	7.3e−08
Date.diffTest	2.0e−03	2.0e−03	9.5e−01	3.5e−01
GenderM	−2.6e−01	1.4e−01	−1.8e+00	7.3e−02
Age	1.0e−03	5.0e−03	1.6e−01	8.8e−01

Anova of the linear model

	Df	Deviance	Resid. Df	Resid. Dev	Pr(>Chi)
NULL	NA	NA	46	18.9	NA
S1.IgG	1	10.1643	45	8.8	1.2e−13
Date.diffTest	1	0.3845	44	8.4	1.5e−01
Gender	1	0.6250	43	7.8	6.6e−02
Age	1	0.0045	42	7.8	8.8e−01

Vaccine: RNA Test: Biosynex

Linear regression of Biosynex, RNA: Test.Result.num ~ S1.IgG + Date.diffTest + Gender + Age, R2=0.759

	Estimate	Std. Error	t value	Pr(>|t|)
(Intercept)	−1.9e−01	2.6e−01	−7.5e−01	4.6e−01
S1.IgG	2.4e−01	2.3e−02	1.0e+01	3.3e−13
Date.diffTest	7.0e−03	2.0e−03	4.1e+00	1.7e−04
GenderM	−2.3e−02	1.1e−01	−2.0e−01	8.4e−01
Age	0.0e+00	4.0e−03	−1.9e−02	9.8e−01

Anova of the linear model

	Df	Deviance	Resid. Df	Resid. Dev	Pr(>Chi)
NULL	NA	NA	46	19.4	NA
S1.IgG	1	1.2e+01	45	7.0	3.6e−26
Date.diffTest	1	2.3e+00	44	4.7	6.1e−06
Gender	1	4.8e−03	43	4.7	8.3e−01
Age	1	4.0e−05	42	4.7	9.8e−01

Vaccine: RNA Test: Dynamiker

Linear regression of Dynamiker, RNA: Test.Result.num ~ S1.IgG + Date.diffTest + Gender + Age, R2=0.035

	Estimate	Std. Error	t value	Pr(>|t|)
(Intercept)	−0.162	0.286	−0.565	0.575
S1.IgG	0.027	0.026	1.032	0.308
Date.diffTest	0.000	0.002	0.114	0.910
GenderM	0.094	0.123	0.761	0.451
Age	0.002	0.004	0.402	0.690

Anova of the linear model

	Df	Deviance	Resid. Df	Resid. Dev	Pr(>Chi)
NULL	NA	NA	46	6.0	NA
S1.IgG	1	0.089	45	5.9	0.42
Date.diffTest	1	0.002	44	5.9	0.90
Gender	1	0.095	43	5.8	0.40
Age	1	0.022	42	5.7	0.69

Vaccine: RNA Test: Cortez

Linear regression of Cortez, RNA: Test.Result.num ~ S1.IgG + Date.diffTest + Gender + Age, R2=0.639

	Estimate	Std. Error	t value	Pr(>|t|)
(Intercept)	−5.2e−01	3.6e−01	−1.4e+00	1.6e−01
S1.IgG	2.5e−01	3.3e−02	7.6e+00	1.9e−09
Date.diffTest	−4.0e−03	2.0e−03	−1.7e+00	9.7e−02
GenderM	−3.6e−02	1.6e−01	−2.3e−01	8.2e−01
Age	9.0e−03	5.0e−03	1.7e+00	9.7e−02

Anova of the linear model

	Df	Deviance	Resid. Df	Resid. Dev	Pr(>Chi)
NULL	NA	NA	46	25.7	NA
S1.IgG	1	1.5e+01	45	10.2	5.8e−17
Date.diffTest	1	3.2e−01	44	9.9	2.3e−01
Gender	1	2.9e−04	43	9.9	9.7e−01
Age	1	6.4e−01	42	9.3	9.0e−02

Vaccine: RNA Test: CTK

Linear regression of CTK, RNA: Test.Result.num ~ S1.IgG + Date.diffTest + Gender + Age, R2=0.57

	Estimate	Std. Error	t value	Pr(>|t|)
(Intercept)	4.7e−01	2.8e−01	1.7e+00	9.5e−02
S1.IgG	1.6e−01	2.5e−02	6.3e+00	1.5e−07
Date.diffTest	3.0e−03	2.0e−03	1.9e+00	6.7e−02
GenderM	−1.6e−01	1.2e−01	−1.3e+00	1.9e−01
Age	4.0e−03	4.0e−03	9.2e−01	3.7e−01

Anova of the linear model

	Df	Deviance	Resid. Df	Resid. Dev	Pr(>Chi)
NULL	NA	NA	46	12.6	NA
S1.IgG	1	6.01	45	6.6	9.4e−12
Date.diffTest	1	0.89	44	5.7	8.6e−03
Gender	1	0.18	43	5.5	2.3e−01
Age	1	0.11	42	5.4	3.6e−01

Vaccine: COVID19+IV

Vaccine: COVID19+IV Test: Abnova

Test results not diverse.

Vaccine: COVID19+IV Test: Nadal

Linear regression of Nadal, COVID19+IV: Test.Result.num ~ S1.IgG + Date.diffTest + Gender + Age, R2=0.509

	Estimate	Std. Error	t value	Pr(>|t|)
(Intercept)	1.97000	0.42300	4.65600	0.00015
S1.IgG	0.06800	0.02900	2.30500	0.03203
Date.diffTest	−0.00200	0.00300	−0.46700	0.64533
GenderM	−0.32100	0.13400	−2.39800	0.02635
Age	−0.00800	0.00900	−0.88800	0.38485

Anova of the linear model

	Df	Deviance	Resid. Df	Resid. Dev	Pr(>Chi)
NULL	NA	NA	24	3.4	NA
S1.IgG	1	0.965	23	2.4	0.00063
Date.diffTest	1	0.235	22	2.2	0.09142
Gender	1	0.444	21	1.7	0.02032
Age	1	0.065	20	1.7	0.37429

Vaccine: COVID19+IV Test: Ring.Biotech

Linear regression of Ring.Biotech, COVID19+IV: Test.Result.num ~ S1.IgG + Date.diffTest + Gender + Age, R2=0.301

	Estimate	Std. Error	t value	Pr(>|t|)
(Intercept)	−0.295	1.053	−0.280	0.782
S1.IgG	0.167	0.073	2.274	0.034
Date.diffTest	0.010	0.009	1.105	0.282
GenderM	−0.579	0.333	−1.736	0.098
Age	0.006	0.023	0.259	0.798

Anova of the linear model

	Df	Deviance	Resid. Df	Resid. Dev	Pr(>Chi)
NULL	NA	NA	24	15	NA
S1.IgG	1	2.704	23	12	0.021
Date.diffTest	1	0.065	22	12	0.721
Gender	1	1.605	21	10	0.076
Age	1	0.034	20	10	0.796

Vaccine: COVID19+IV Test: Wondfo

Linear regression of Wondfo, COVID19+IV: Test.Result.num ~ S1.IgG + Date.diffTest + Gender + Age, R2=0.552

	Estimate	Std. Error	t value	Pr(>|t|)
(Intercept)	1.7910	0.8180	2.1910	0.0405
S1.IgG	0.1810	0.0570	3.1720	0.0048
Date.diffTest	−0.0030	0.0070	−0.3750	0.7117
GenderM	−0.4430	0.2590	−1.7140	0.1021
Age	−0.0330	0.0180	−1.8550	0.0783

Anova of the linear model

	Df	Deviance	Resid. Df	Resid. Dev	Pr(>Chi)
NULL	NA	NA	24	13.8	NA
S1.IgG	1	5.36	23	8.4	3.1e−05
Date.diffTest	1	0.45	22	8.0	2.3e−01
Gender	1	0.73	21	7.2	1.3e−01
Age	1	1.06	20	6.2	6.4e−02

Vaccine: COVID19+IV Test: Labnovation

Linear regression of Labnovation, COVID19+IV: Test.Result.num ~ S1.IgG + Date.diffTest + Gender + Age, R2=0.27

	Estimate	Std. Error	t value	Pr(>|t|)
(Intercept)	1.661	0.965	1.721	0.101
S1.IgG	0.148	0.067	2.207	0.039
Date.diffTest	0.003	0.008	0.348	0.731
GenderM	−0.041	0.305	−0.134	0.895
Age	−0.030	0.021	−1.424	0.170

Anova of the linear model

	Df	Deviance	Resid. Df	Resid. Dev	Pr(>Chi)
NULL	NA	NA	24	11.8	NA
S1.IgG	1	2.2e+00	23	9.5	0.022
Date.diffTest	1	6.0e−02	22	9.5	0.710
Gender	1	4.3e−05	21	9.5	0.992
Age	1	8.7e−01	20	8.6	0.155

Vaccine: COVID19+IV Test: Biosynex

Linear regression of Biosynex, COVID19+IV: Test.Result.num ~ S1.IgG + Date.diffTest + Gender + Age, R2=0.553

	Estimate	Std. Error	t value	Pr(>|t|)
(Intercept)	0.0590	0.7700	0.0770	0.9394
S1.IgG	0.2430	0.0540	4.5320	0.0002
Date.diffTest	0.0070	0.0060	1.1580	0.2606
GenderM	−0.3170	0.2440	−1.2990	0.2086
Age	−0.0040	0.0170	−0.2560	0.8005

Anova of the linear model

	Df	Deviance	Resid. Df	Resid. Dev	Pr(>Chi)
NULL	NA	NA	24	12.2	NA
S1.IgG	1	6.184	23	6.1	2.0e−06
Date.diffTest	1	0.117	22	5.9	5.1e−01
Gender	1	0.448	21	5.5	2.0e−01
Age	1	0.018	20	5.5	8.0e−01

Vaccine: COVID19+IV Test: Dynamiker

Linear regression of Dynamiker, COVID19+IV: Test.Result.num ~ S1.IgG + Date.diffTest + Gender + Age, R2=0.532

	Estimate	Std. Error	t value	Pr(>|t|)
(Intercept)	0.6810	0.9170	0.7420	0.4665
S1.IgG	0.1860	0.0640	2.9070	0.0087
Date.diffTest	0.0020	0.0080	0.2290	0.8214
GenderM	−0.7880	0.2900	−2.7130	0.0134
Age	−0.0060	0.0200	−0.3260	0.7477

Anova of the linear model

	Df	Deviance	Resid. Df	Resid. Dev	Pr(>Chi)
NULL	NA	NA	24	16.6	NA
S1.IgG	1	5.493	23	11.1	0.00017
Date.diffTest	1	0.459	22	10.6	0.27671
Gender	1	2.814	21	7.8	0.00706
Age	1	0.041	20	7.8	0.74429

Vaccine: COVID19+IV Test: Cortez

Linear regression of Cortez, COVID19+IV: Test.Result.num ~ S1.IgG + Date.diffTest + Gender + Age, R2=0.23

	Estimate	Std. Error	t value	Pr(>|t|)
(Intercept)	2.32200	0.53000	4.38100	0.00029
S1.IgG	−0.00700	0.03700	−0.19000	0.85098
Date.diffTest	−0.01000	0.00400	−2.18900	0.04062
GenderM	0.12600	0.16800	0.75000	0.46173
Age	0.00300	0.01100	0.22600	0.82383

Anova of the linear model

	Df	Deviance	Resid. Df	Resid. Dev	Pr(>Chi)
NULL	NA	NA	24	3.4	NA
S1.IgG	1	0.1366	23	3.2	0.304
Date.diffTest	1	0.5588	22	2.7	0.038
Gender	1	0.0692	21	2.6	0.465
Age	1	0.0066	20	2.6	0.822

Vaccine: COVID19+IV Test: CTK

Linear regression of CTK, COVID19+IV: Test.Result.num ~ S1.IgG + Date.diffTest + Gender + Age, R2=0.498

	Estimate	Std. Error	t value	Pr(>|t|)
(Intercept)	1.85900	0.46700	3.98400	0.00073
S1.IgG	0.09400	0.03300	2.87800	0.00930
Date.diffTest	−0.00100	0.00400	−0.36500	0.71875
GenderM	−0.24200	0.14800	−1.64100	0.11647
Age	−0.01100	0.01000	−1.14300	0.26645

Anova of the linear model

	Df	Deviance	Resid. Df	Resid. Dev	Pr(>Chi)
NULL	NA	NA	24	4.0	NA
S1.IgG	1	1.48	23	2.5	0.00012
Date.diffTest	1	0.14	22	2.4	0.23526
Gender	1	0.24	21	2.1	0.12521
Age	1	0.13	20	2.0	0.25295

Vaccine: IV

Vaccine: IV Test: Abnova

Test results not diverse.

Vaccine: IV Test: Nadal

Linear regression of Nadal, IV: Test.Result.num ~ S1.IgG + Date.diffTest + Gender + Age, R2=0.728

	Estimate	Std. Error	t value	Pr(>|t|)
(Intercept)	−8.2e−01	5.2e−01	−1.6e+00	1.3e−01
S1.IgG	4.0e−01	4.2e−02	9.4e+00	6.1e−11
Date.diffTest	5.0e−03	6.0e−03	8.2e−01	4.2e−01
GenderM	−2.5e−01	1.4e−01	−1.8e+00	8.0e−02
Age	7.0e−03	8.0e−03	9.0e−01	3.7e−01

Anova of the linear model

	Df	Deviance	Resid. Df	Resid. Dev	Pr(>Chi)
NULL	NA	NA	38	23.6	NA
S1.IgG	1	16.28	37	7.3	1.5e−20
Date.diffTest	1	0.14	36	7.2	3.8e−01
Gender	1	0.61	35	6.6	7.2e−02
Age	1	0.15	34	6.4	3.7e−01

Vaccine: IV Test: Ring.Biotech

Linear regression of Ring.Biotech, IV: Test.Result.num ~ S1.IgG + Date.diffTest + Gender + Age, R2=0.352

	Estimate	Std. Error	t value	Pr(>|t|)
(Intercept)	0.06600	0.45900	0.14300	0.88738
S1.IgG	0.13600	0.03700	3.66000	0.00085
Date.diffTest	−0.00100	0.00500	−0.23200	0.81813
GenderM	0.16700	0.12200	1.36800	0.18023
Age	−0.00600	0.00700	−0.86800	0.39148

Anova of the linear model

	Df	Deviance	Resid. Df	Resid. Dev	Pr(>Chi)
NULL	NA	NA	38	7.6	NA
S1.IgG	1	2.280	37	5.3	7.2e−05
Date.diffTest	1	0.011	36	5.3	7.8e−01
Gender	1	0.269	35	5.0	1.7e−01
Age	1	0.109	34	4.9	3.9e−01

Vaccine: IV Test: Wondfo

Linear regression of Wondfo, IV: Test.Result.num ~ S1.IgG + Date.diffTest + Gender + Age, R2=0.3

	Estimate	Std. Error	t value	Pr(>|t|)
(Intercept)	−0.3300	0.6460	−0.5100	0.6131
S1.IgG	0.1730	0.0520	3.3070	0.0022
Date.diffTest	−0.0040	0.0070	−0.5080	0.6146
GenderM	0.0070	0.1720	0.0430	0.9662
Age	0.0130	0.0100	1.3730	0.1786

Anova of the linear model

	Df	Deviance	Resid. Df	Resid. Dev	Pr(>Chi)
NULL	NA	NA	38	13.9	NA
S1.IgG	1	3.56773	37	10.3	0.00041
Date.diffTest	1	0.06472	36	10.3	0.63431
Gender	1	0.00074	35	10.3	0.95936
Age	1	0.53951	34	9.7	0.16962

Vaccine: IV Test: Labnovation

Linear regression of Labnovation, IV: Test.Result.num ~ S1.IgG + Date.diffTest + Gender + Age, R2=0.708

	Estimate	Std. Error	t value	Pr(>|t|)
(Intercept)	4.0e−01	5.7e−01	7.0e−01	4.9e−01
S1.IgG	3.8e−01	4.6e−02	8.4e+00	8.9e−10
Date.diffTest	−9.0e−03	6.0e−03	−1.4e+00	1.7e−01
GenderM	−2.0e−01	1.5e−01	−1.3e+00	1.9e−01
Age	3.0e−03	9.0e−03	3.3e−01	7.4e−01

Anova of the linear model

	Df	Deviance	Resid. Df	Resid. Dev	Pr(>Chi)
NULL	NA	NA	38	25.6	NA
S1.IgG	1	17.280	37	8.3	7.9e−19
Date.diffTest	1	0.412	36	7.9	1.7e−01
Gender	1	0.392	35	7.5	1.8e−01
Age	1	0.024	34	7.5	7.4e−01

Vaccine: IV Test: Biosynex

Linear regression of Biosynex, IV: Test.Result.num ~ S1.IgG + Date.diffTest + Gender + Age, R2=0.724

	Estimate	Std. Error	t value	Pr(>|t|)
(Intercept)	−1.2e+00	4.4e−01	−2.7e+00	1.2e−02
S1.IgG	3.3e−01	3.6e−02	9.4e+00	6.2e−11
Date.diffTest	8.0e−03	5.0e−03	1.7e+00	1.0e−01
GenderM	−8.1e−02	1.2e−01	−6.9e−01	4.9e−01
Age	8.0e−03	7.0e−03	1.2e+00	2.3e−01

Anova of the linear model

	Df	Deviance	Resid. Df	Resid. Dev	Pr(>Chi)
NULL	NA	NA	38	16.3	NA
S1.IgG	1	11.159	37	5.1	4.2e−20
Date.diffTest	1	0.393	36	4.8	8.5e−02
Gender	1	0.062	35	4.7	4.9e−01
Age	1	0.194	34	4.5	2.3e−01

Vaccine: IV Test: Dynamiker

Linear regression of Dynamiker, IV: Test.Result.num ~ S1.IgG + Date.diffTest + Gender + Age, R2=0.458

	Estimate	Std. Error	t value	Pr(>|t|)
(Intercept)	−6.1e−01	5.4e−01	−1.1e+00	2.6e−01
S1.IgG	2.2e−01	4.3e−02	5.1e+00	1.4e−05
Date.diffTest	3.0e−03	6.0e−03	4.6e−01	6.5e−01
GenderM	1.5e−01	1.4e−01	1.0e+00	3.0e−01
Age	3.0e−03	8.0e−03	4.1e−01	6.9e−01

Anova of the linear model

	Df	Deviance	Resid. Df	Resid. Dev	Pr(>Chi)
NULL	NA	NA	38	12.4	NA
S1.IgG	1	5.375	37	7.0	1.7e−07
Date.diffTest	1	0.041	36	6.9	6.5e−01
Gender	1	0.216	35	6.7	2.9e−01
Age	1	0.033	34	6.7	6.8e−01

Vaccine: IV Test: Cortez

Linear regression of Cortez, IV: Test.Result.num ~ S1.IgG + Date.diffTest + Gender + Age, R2=0.604

	Estimate	Std. Error	t value	Pr(>|t|)
(Intercept)	1.8e+00	6.0e−01	3.0e+00	5.7e−03
S1.IgG	2.6e−01	4.8e−02	5.3e+00	7.5e−06
Date.diffTest	−2.3e−02	7.0e−03	−3.5e+00	1.2e−03
GenderM	−1.2e−01	1.6e−01	−7.5e−01	4.6e−01
Age	1.0e−03	9.0e−03	8.8e−02	9.3e−01

Anova of the linear model

	Df	Deviance	Resid. Df	Resid. Dev	Pr(>Chi)
NULL	NA	NA	38	21.0	NA
S1.IgG	1	9.4953	37	11.5	4.5e−10
Date.diffTest	1	3.0393	36	8.4	4.2e−04
Gender	1	0.1377	35	8.3	4.5e−01
Age	1	0.0019	34	8.3	9.3e−01

Vaccine: IV Test: CTK

Linear regression of CTK, IV: Test.Result.num ~ S1.IgG + Date.diffTest + Gender + Age, R2=0.781

	Estimate	Std. Error	t value	Pr(>|t|)
(Intercept)	−9.8e−01	4.3e−01	−2.3e+00	2.9e−02
S1.IgG	3.8e−01	3.5e−02	1.1e+01	1.3e−12
Date.diffTest	6.0e−03	5.0e−03	1.3e+00	2.1e−01
GenderM	−9.0e−02	1.1e−01	−7.8e−01	4.4e−01
Age	8.0e−03	6.0e−03	1.3e+00	2.0e−01

Anova of the linear model

	Df	Deviance	Resid. Df	Resid. Dev	Pr(>Chi)
NULL	NA	NA	38	19.7	NA
S1.IgG	1	14.878	37	4.8	2.2e−27
Date.diffTest	1	0.220	36	4.6	1.9e−01
Gender	1	0.076	35	4.5	4.4e−01
Age	1	0.215	34	4.3	1.9e−01

Vaccine: COVID19

Vaccine: COVID19 Test: Abnova

Test results not diverse.

Vaccine: COVID19 Test: Nadal

Linear regression of Nadal, COVID19: Test.Result.num ~ S1.IgG + Date.diffTest + Gender + Age, R2=0.537

	Estimate	Std. Error	t value	Pr(>|t|)
(Intercept)	8.7e−01	6.1e−01	1.4e+00	1.7e−01
S1.IgG	1.7e−01	3.1e−02	5.3e+00	9.9e−06
Date.diffTest	−6.0e−03	6.0e−03	−9.2e−01	3.7e−01
GenderM	3.0e−01	2.7e−01	1.1e+00	2.8e−01
Age	−4.0e−03	9.0e−03	−5.0e−01	6.2e−01

Anova of the linear model

	Df	Deviance	Resid. Df	Resid. Dev	Pr(>Chi)
NULL	NA	NA	33	22	NA
S1.IgG	1	10.887	32	11	2.8e−08
Date.diffTest	1	0.440	31	11	2.6e−01
Gender	1	0.470	30	10	2.5e−01
Age	1	0.087	29	10	6.2e−01

Vaccine: COVID19 Test: Ring.Biotech

Linear regression of Ring.Biotech, COVID19: Test.Result.num ~ S1.IgG + Date.diffTest + Gender + Age, R2=0.296

	Estimate	Std. Error	t value	Pr(>|t|)
(Intercept)	1.723	0.832	2.070	0.047
S1.IgG	0.115	0.042	2.734	0.011
Date.diffTest	−0.014	0.008	−1.619	0.116
GenderM	−0.194	0.368	−0.527	0.602
Age	−0.002	0.012	−0.186	0.854

Anova of the linear model

	Df	Deviance	Resid. Df	Resid. Dev	Pr(>Chi)
NULL	NA	NA	33	27	NA
S1.IgG	1	6.102	32	21	0.0021
Date.diffTest	1	1.584	31	19	0.1174
Gender	1	0.169	30	19	0.6093
Age	1	0.022	29	19	0.8526

Vaccine: COVID19 Test: Wondfo

Linear regression of Wondfo, COVID19: Test.Result.num ~ S1.IgG + Date.diffTest + Gender + Age, R2=0.083

	Estimate	Std. Error	t value	Pr(>|t|)
(Intercept)	0.235	0.776	0.303	0.764
S1.IgG	0.007	0.039	0.173	0.864
Date.diffTest	−0.004	0.008	−0.570	0.573
GenderM	0.048	0.343	0.141	0.889
Age	0.016	0.011	1.493	0.146

Anova of the linear model

	Df	Deviance	Resid. Df	Resid. Dev	Pr(>Chi)
NULL	NA	NA	33	18	NA
S1.IgG	1	6.2e−02	32	18	0.74
Date.diffTest	1	1.6e−01	31	18	0.59
Gender	1	6.3e−06	30	18	1.00
Age	1	1.3e+00	29	16	0.14

Vaccine: COVID19 Test: Labnovation

Linear regression of Labnovation, COVID19: Test.Result.num ~ S1.IgG + Date.diffTest + Gender + Age, R2=0.247

	Estimate	Std. Error	t value	Pr(>|t|)
(Intercept)	1.8700	0.6720	2.7820	0.0094
S1.IgG	0.0870	0.0340	2.5560	0.0161
Date.diffTest	−0.0080	0.0070	−1.1450	0.2617
GenderM	0.0440	0.2970	0.1480	0.8832
Age	−0.0050	0.0090	−0.5390	0.5941

Anova of the linear model

	Df	Deviance	Resid. Df	Resid. Dev	Pr(>Chi)
NULL	NA	NA	33	16	NA
S1.IgG	1	3.259	32	13	0.0054
Date.diffTest	1	0.610	31	12	0.2289
Gender	1	0.017	30	12	0.8406
Age	1	0.122	29	12	0.5900

Vaccine: COVID19 Test: Biosynex

Linear regression of Biosynex, COVID19: Test.Result.num ~ S1.IgG + Date.diffTest + Gender + Age, R2=0.484

	Estimate	Std. Error	t value	Pr(>|t|)
(Intercept)	3.0e−01	7.0e−01	4.4e−01	6.7e−01
S1.IgG	1.7e−01	3.5e−02	5.0e+00	2.8e−05
Date.diffTest	−1.0e−03	7.0e−03	−1.8e−01	8.6e−01
GenderM	3.8e−01	3.1e−01	1.2e+00	2.3e−01
Age	0.0e+00	1.0e−02	−2.5e−02	9.8e−01

Anova of the linear model

	Df	Deviance	Resid. Df	Resid. Dev	Pr(>Chi)
NULL	NA	NA	33	26	NA
S1.IgG	1	1.2e+01	32	14	4.3e−07
Date.diffTest	1	6.1e−02	31	14	7.1e−01
Gender	1	6.9e−01	30	13	2.2e−01
Age	1	2.7e−04	29	13	9.8e−01

Vaccine: COVID19 Test: Dynamiker

Linear regression of Dynamiker, COVID19: Test.Result.num ~ S1.IgG + Date.diffTest + Gender + Age, R2=0.254

	Estimate	Std. Error	t value	Pr(>|t|)
(Intercept)	0.8390	0.7960	1.0540	0.3007
S1.IgG	0.1130	0.0400	2.8110	0.0088
Date.diffTest	−0.0050	0.0080	−0.6810	0.5014
GenderM	0.2120	0.3520	0.6040	0.5505
Age	−0.0030	0.0110	−0.2730	0.7865

Anova of the linear model

	Df	Deviance	Resid. Df	Resid. Dev	Pr(>Chi)
NULL	NA	NA	33	23	NA
S1.IgG	1	5.176	32	18	0.0031
Date.diffTest	1	0.371	31	17	0.4283
Gender	1	0.237	30	17	0.5265
Age	1	0.044	29	17	0.7846

Vaccine: COVID19 Test: Cortez

Linear regression of Cortez, COVID19: Test.Result.num ~ S1.IgG + Date.diffTest + Gender + Age, R2=0.307

	Estimate	Std. Error	t value	Pr(>|t|)
(Intercept)	1.3560	0.6860	1.9770	0.0576
S1.IgG	0.1120	0.0350	3.2230	0.0031
Date.diffTest	−0.0060	0.0070	−0.9110	0.3698
GenderM	−0.1560	0.3030	−0.5150	0.6107
Age	−0.0010	0.0100	−0.1090	0.9139

Anova of the linear model

	Df	Deviance	Resid. Df	Resid. Dev	Pr(>Chi)
NULL	NA	NA	33	18	NA
S1.IgG	1	5.2157	32	13	0.00057
Date.diffTest	1	0.3157	31	13	0.39650
Gender	1	0.1126	30	13	0.61255
Age	1	0.0052	29	13	0.91320

Vaccine: COVID19 Test: CTK

Linear regression of CTK, COVID19: Test.Result.num ~ S1.IgG + Date.diffTest + Gender + Age, R2=0.463

	Estimate	Std. Error	t value	Pr(>|t|)
(Intercept)	3.6e−01	7.5e−01	4.8e−01	6.3e−01
S1.IgG	1.8e−01	3.8e−02	4.9e+00	3.8e−05
Date.diffTest	−2.0e−03	8.0e−03	−2.1e−01	8.4e−01
GenderM	1.4e−01	3.3e−01	4.2e−01	6.8e−01
Age	0.0e+00	1.1e−02	−3.8e−02	9.7e−01

Anova of the linear model

	Df	Deviance	Resid. Df	Resid. Dev	Pr(>Chi)
NULL	NA	NA	33	28	NA
S1.IgG	1	1.3e+01	32	15	6.7e−07
Date.diffTest	1	4.0e−02	31	15	7.8e−01
Gender	1	9.4e−02	30	15	6.7e−01
Age	1	7.4e−04	29	15	9.7e−01

Supplementary File S2: Other Variable Effect

Gender

A B

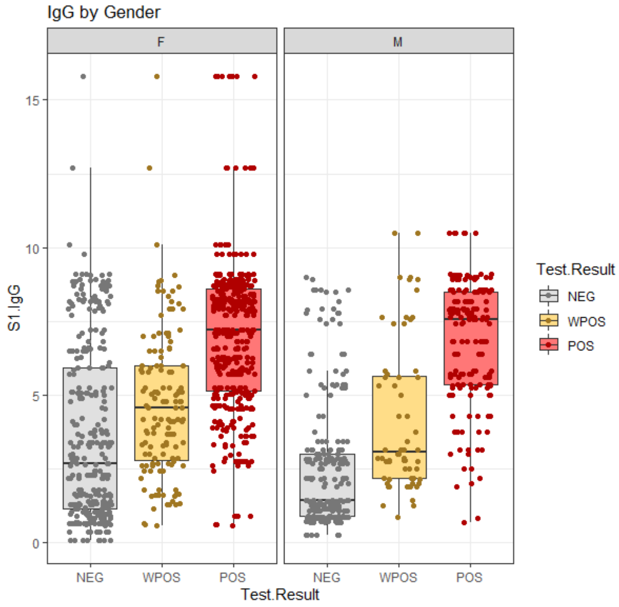

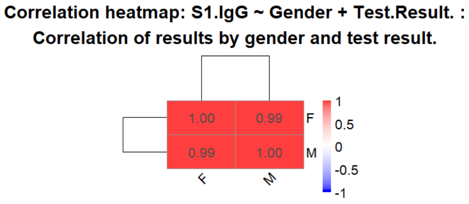

Figure S1: This dataset is not balanced for gender, as there are roughly two times more females than males. Regardless this imbalance, we observed no particular difference between the S1.IgG concentration and the test response. Figure S.1.A displays the overall trend between genders, which is further proved by the correlation analysis in the figure S.1.A. Again, even when separated by tests, vaccines and immune type, none of the regression analyzes found gender as a significant variable.

Age by groups

A

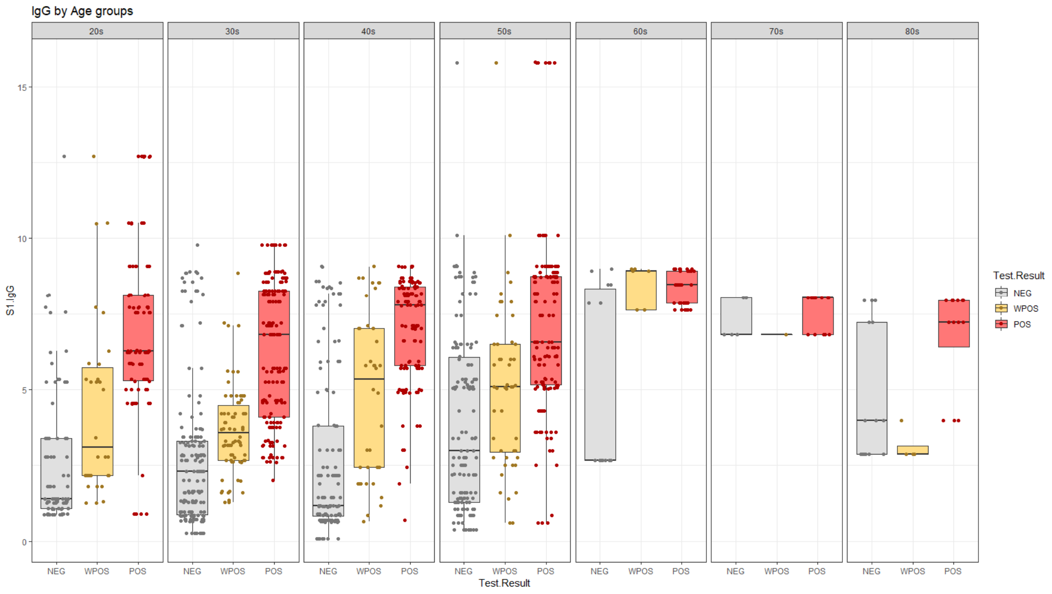

B

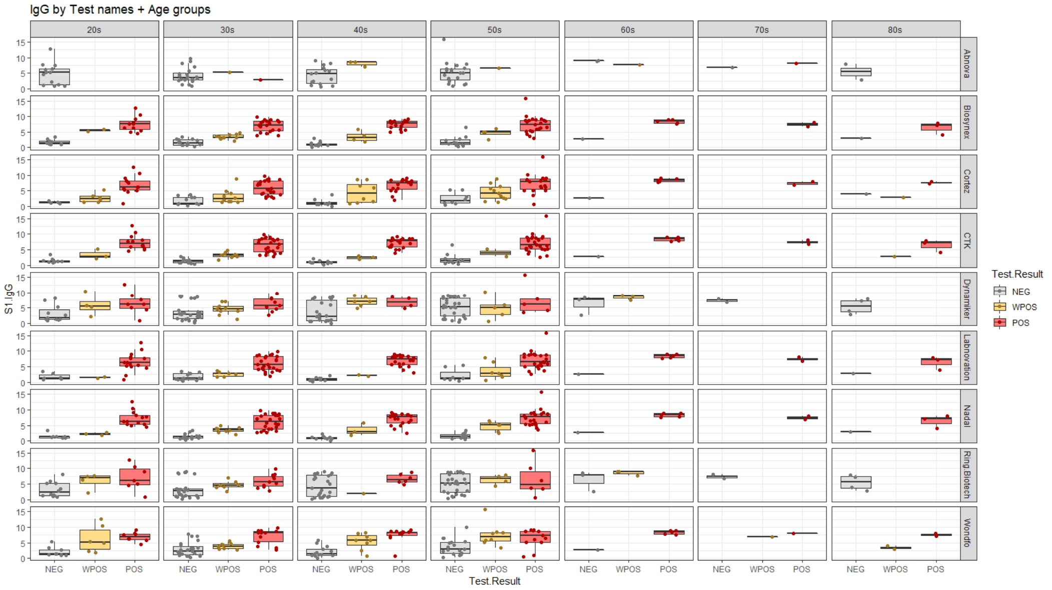

C

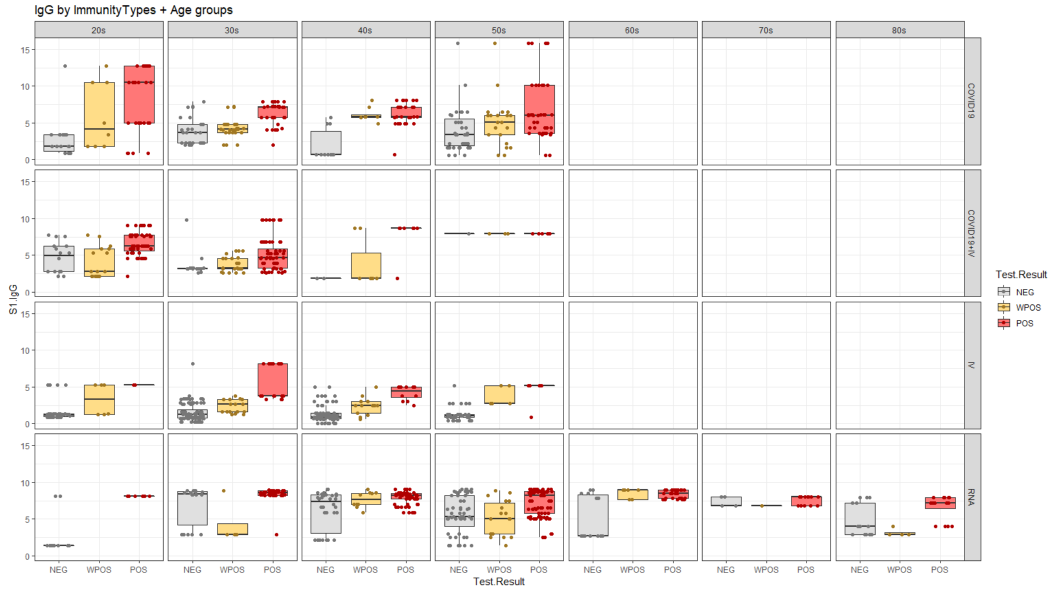

Figure S2: A, results separated by age groups. **B**, results separated by age groups and by test. **C**, results separated by age groups and immune type.

Time differential

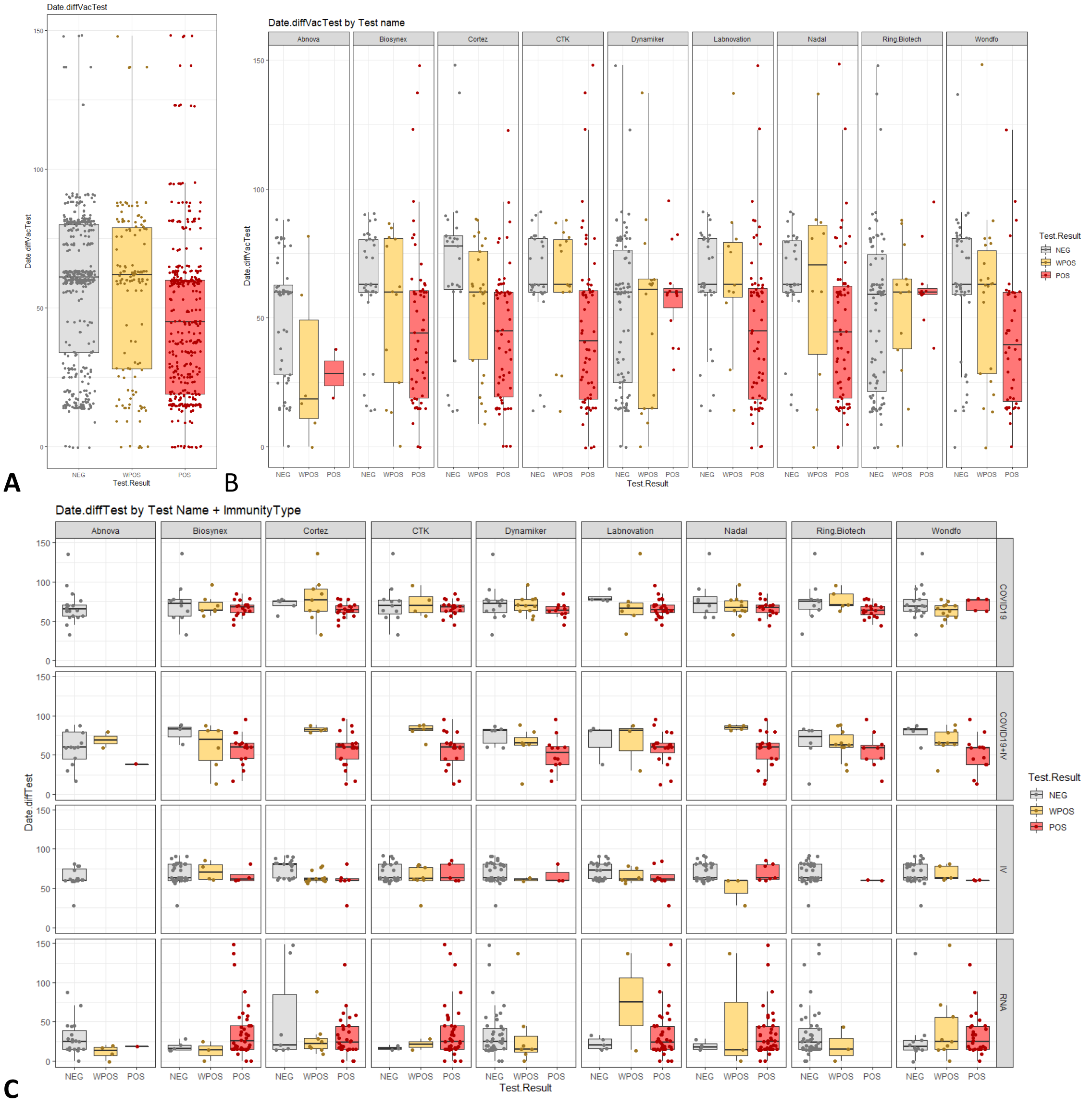

Figure S3: A brief look at the tests results and their respective different in time between the test and the vaccination or infection allows is to pick some form of trends (**A**). This logical trend shows that time has somewhat a negative impact on the quantity of S1 IgG. When looking at test specific (**B**), it is clear that some test are more sensitive to the effect, but these differences can also be explained by the performance of the tests depending on the subjects. Once separated by immunity type (**C**), it becomes clearer that the effect of time is negligible in most cases and questionable in few. This is consistent with the regression results, in which no strong effect of time was measured. Two cases or weak effect were identified, but cross checking with this figure clearly appears to be unwanted bias introduced in the subject sampling. We pushed the analysis a little more and observed that even when taking into account only S1.IgG and time all the while separating the data by age groups, no trend is remotely observable.

Confirmation by regression modeling

To confirm the linear fitting of the test results against the S1.IgG and other variables, Gaussian distribution was chosen with logit identity to build normal regression model. To validate any potential significant variable, chi-squared test (anova) is performed to validate the goodness of fit.

Essentially, these steps allowed to define whether there is a linear response between Test results (as numeric values: Negative=0, weak positive=1 and positive=2), measured IgG concentration, and the other measured variables (date, gender and age).

By separating the tests by Test brands and by type of vaccine, it is possible to validate whether in the given condition the test result is valid and if any of the other variables have an impact.

All the test tables are shown the supplementary file X. Two rounds of models were tested, separated by Vaccine and by immune type. A summary table for each round is shown in the figures 4A and 4C, and their respective linearity score (R^2) in the figures 4B and 4D. In the first set it is possible to evaluate each vaccine, the second set allows to evaluate the effect of IV vaccine with or with prior infection. Logically, if no significant variable contributing to the linearity is found, R^2 score is very low. In the vaccine round, only S1.IgG was found significant in all the tests, while in the immune type round, time was found in two cases as significant contributor. However, these two cases are questionable results as the significance is rather low, and the number of data points is rather limited. The non vaccinated response is expectably the same between the two groupings, but Moderna and Pfiser as mRNA vaccine do not return the significant results with the same tests.

A

	Pfizer	Moderna	SinoVac	None
Abnova				
Nadal		S1.IgG	S1.IgG	S1.IgG
Ring.Biotec			S1.IgG	
Wondfo	S1.IgG	S1.IgG	S1.IgG	
Labnovation			S1.IgG	
Biosynex	S1.IgG		S1.IgG	S1.IgG
Dynamiker			S1.IgG	
Cortez		S1.IgG	S1.IgG	S1.IgG
CTK			S1.IgG	S1.IgG

B

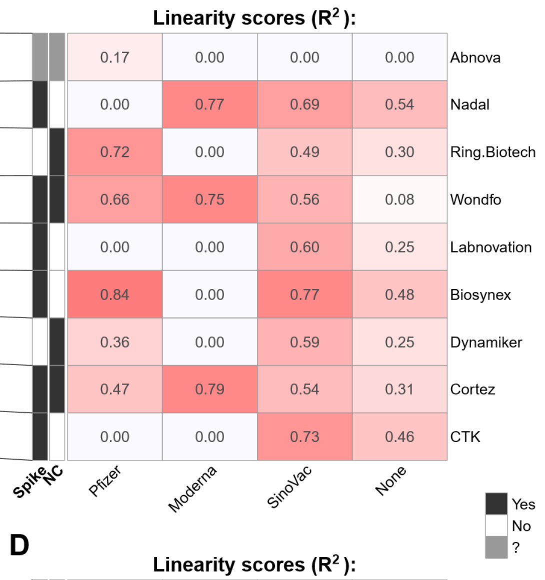

C

	RNA	COVID19 +IV	IV	COVID19
Abnova				
Nadal	S1.IgG		S1.IgG	S1.IgG
Ring.Biotec			S1.IgG	
Wondfo	S1.IgG	S1.IgG	S1.IgG	
Labnovation	S1.IgG		S1.IgG	
Biosynex	S1.IgG Date.diffTest	S1.IgG	S1.IgG	S1.IgG
Dynamiker			S1.IgG	
Cortez	S1.IgG		S1.IgG, Date.diffTes	S1.IgG
CTK	S1.IgG		S1.IgG	S1.IgG

D

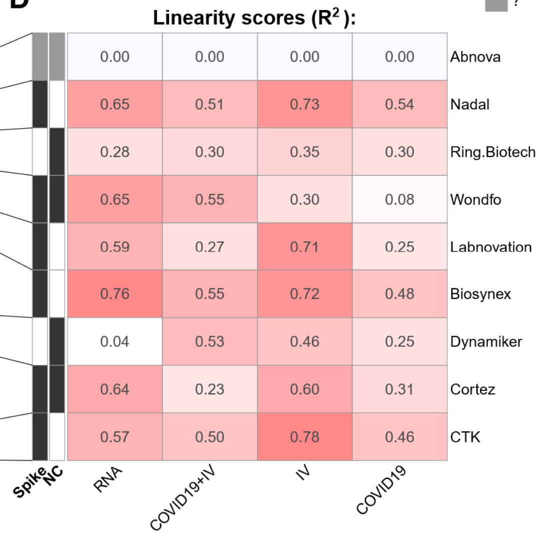

Figure S4: *Tables contain the significant variables resulting in linear response of test results. Empty cell means either no linearity was found or no significant variable. The corresponding R^2 value of each test is shown in the heatmaps, 1 being perfect linearity, 0 test could not be performed.*
